# A Review of the Prevalence of Illicit Substance Use in Solid-Organ Transplant Candidates and the Effects of Illicit Substance Use on Solid-Organ Transplant Treatment Outcomes

**DOI:** 10.7759/cureus.8986

**Published:** 2020-07-03

**Authors:** Pradipta Majumder, Siddharth Sarkar

**Affiliations:** 1 Psychiatry, WellSpan Health, York, USA; 2 Addiction, All India Institute of Medical Sciences, New Delhi, IND

**Keywords:** solid organ transplant, substance use, outcome

## Abstract

Solid-organ transplantation is one of the significant advances in the medical field that have improved the quality of life and survival rates of numerous patients with end-organ dysfunction. Substance use is a common condition of individuals who are in need of solid-organ transplantation. The implications of substance use on solid-organ transplants are gaining increasing attention over the past decade. The current review seeks to explore the prevalence rate of illicit substance use among those who receive solid-organ transplantation (pre and post-transplant) and whether illicit substance use before solid-organ transplantation affects the outcome of solid-organ transplants. We searched the Medline database for all the articles available in English on the prevalence of substance use in the context of solid-organ transplant and the effect on outcome measures. We found 21 relevant articles. It appears that substance use is fairly common among solid-organ transplant candidates, with cannabis being the most common substance of abuse. A heterogeneous sample precludes the drawing of a clear-cut conclusion. But it appears that substance use may affect various outcomes of solid-organ transplants. The existing literature may not be sufficient to adequately assess the risk but limited evidence indicates that illicit substance use, particularly cannabis use, may not affect the overall survival following a solid-organ transplant.

## Introduction and background

Since its inception in the twentieth century, solid-organ transplantation has been increasingly used for the treatment of end-organ damage and has significantly improved the life expectancy and quality of life of the recipients [1-2]. With the advent of newer technologies, surgical skills, and newer treatment options, the rate of solid-organ transplantation has increased significantly over the past decades. In 2005, the total number of solid-organ transplantation was 28,119, whereas, in 2019, the number was 39,719 [3]. However, there is a significant mismatch between organ donors and the number of people requiring a transplant. As of March 2020, there are more than 112,000 candidates in the organ transplant waiting list with limited donors [4]. The mismatch, in turn, leads to careful consideration of the allocation of resources and the selection of recipients of solid-organ transplants.

Alcohol or illicit substances are implicated in the causal pathway of end-organ damage, and substance users are more likely to require liver transplantation [5]. However, substance users or former substance users often do not get accepted in the recipient lists. For example, the International Society of Heart and Lung Transplantation recommends rendering patients with active alcohol and illicit substance use as unsuitable for heart transplantation because of concern of a risk of poor outcome after such transplantation [6-7]. There were debates in the past on the priority of individuals with alcohol-induced end-stage liver disease to receive liver transplantation [8-9]. There is a great deal of interest in the research on the implications of substance use on solid-organ transplant outcomes. In general, abstinence from alcohol and other substances is required for receiving a solid-organ transplant, but in some centers, complete abstinence from alcohol is not a necessary criterion before transplantation when liver disease is not related to alcohol use disorder and when alcohol consumption is within the recommended limits for the general population [10]. Also, in July 2015, the California Medical Cannabis Organ Transplant Act prohibits a denial of transplantation based solely on the prospective recipient’s marijuana use or solely upon a positive test for the use of medical marijuana [11]. Despite a trend of a broader inclusion of individuals with a history of substance use in the transplant list, there are always concerns about the risk of relapse to substance use, poor treatment adherence in the post-transplant period, and a poor outcome of a solid0organ transplant.

The bulk of the substance use literature has focused on alcohol use in the context of solid-organ transplants, particularly in the association of liver transplantation. However, there has been much less effort to review the data on illicit drug use. Such substance use may not only influence the consideration of transplantation but may also affect the outcomes. In our knowledge, there was no review work done on the relapse rate of illicit substances in post-transplant patients since 2008 [12]. Hence, the current review aimed to investigate the prevalence rate of illicit substance use among those who receive solid-organ transplantation (pre and post-transplant) and whether illicit substance use before solid-organ transplantation predicts poorer outcomes as compared to non-users of illicit drugs.

## Review

Methodology

The review aimed at collating studies that provided information about illicit substance use among patients who were planned for solid-organ transplantation or had received such transplantation. For our review, we only selected PubMed indexed peer-reviewed journal articles. We used the Medline database as the search engine. We kept a broad inclusion criterion to ensure flexibility and to ensure incorporation of as many studies as possible. We included observational studies, case series, and quantitative and qualitative data-based studies. We included opioids, cocaine, cannabis, methamphetamine, LSD, ecstasy, amphetamines, and others as illicit substances. We have also included studies that have used the term "substance users" without further clarifying the subcategories in the present review. We selected the heart, kidney, lung, pancreas, and liver transplant and included only those studies that were in the English language and involved adults. The keywords used were “solid organ transplant, and illicit”, “solid organ transplant, Illicit substance use”, “substance use, transplant”, “substance use, solid organ transplant”, “transplant, illicit drug use”, “substance use relapse, transplant”, “illicit drug use, pre and post transplant”, “pre and post transplant, substance use”, and “post transplant, substance use” in varying combinations. Additional search items, including the names of each substance and each solid organ transplant, were also undertaken. The searches were done by one of the investigators (PM). Additional articles were identified using cross-references. We did not include any unpublished material as part of this study. Identified abstracts were screened, and full texts of relevant articles were obtained. Data about the year of publication, sample size, sample population, types of illicit substances reported, the occurrence rate of illicit substance use, and outcomes like survival of graft, peri-operative complications, relapse to substance use, and deaths were extracted. We presented the outcome measures of the included studies in two categories: studies on the prevalence of illicit substance use and studies on the implications of substance use in the post-transplant period. Due to the heterogeneity of studies and outcome parameters/methods of assessment of occurrence rates, we did not attempt a quantitative synthesis with the data.

Results

Using the key items in various combinations, we retrieved 1037 articles following the Preferred Reporting Items for Systematic Reviews and Meta-Analyses (PRISMA) guidelines (as shown in the Appendix). After the removal of duplicates, there were 970 articles left. Among the 970 articles, 583 were screened based upon the titles and abstracts, and 55 articles were assessed for eligibility. We excluded 34 articles for not addressing the aim of our review, for primarily addressing the implications of alcohol use, for addressing the issues on donors, and for primarily investigating prescription opioids on solid-organ transplants. Finally, 21 articles were included in this review.

Characteristics of the included studies

We found wide variations of the methodologies used in the included studies. Some studies investigated patients who are still in the process of evaluation for the suitability of a solid-organ transplant [10,13-14]. Some have included patients who are already listed for the transplant [15], whereas others assessed the patients in the post-transplant period [16-18]. Most of the studies included in this research were retrospective studies [13-14,17]. We found only one study that utilized prospective study design [19]. The majority of the studies have reviewed charts and clinical data from the transplant registry of universities or clinics [15-16,18,20-22]. One study utilized the national database linking national kidney transplant records with Medicare claims [23]. The study populations were different from each other in terms of associated conditions or diseases. For example, Robaeys et al. (2009) included all Hepatitis C virus (HCV) positive patients who underwent a liver transplant and compared the outcome between individuals with a history of intravenous drug user (IVDU) and those without a history of IVDU [20]. Studies also investigated the use of illicit substances in the context of a primary alcohol use disorder [22,24] or injecting drug use [16]. Some of the studies utilized a survey method; for example, Sacco et al. (2018) developed a comprehensive survey to examine substance use and psychosocial and mental health variables in a group of individuals who received a liver transplant and were diagnosed with alcohol-related liver disease. The participants were emailed and then contacted by phone calls, inviting them to participate in the study [24]. Similarly, Lamba (2012) also used a mailed questionnaire [25]. The other studies employed a cross-sectional assessment of data [10,26]. One study also used incentives for participating in the study [24]. Only one study specified if the participants underwent only one type of transplant vs. multi-organ transplant and or if the transplant was the first transplant vs. repeat transplant [20]. The sample sizes of some of the studies varied widely from 27 to 52,689 [8,23].

Illicit substance use among solid-organ transplant candidates

Thirteen studies reported the rates of illicit substance use in solid-organ transplant candidates (Table 1). Seven studies reviewed retrospective data [13-18,22], three studies used cross-sectional assessment [10,25-26], one study analyzed the national transplant registry [23], one study utilized prospective study design [19], and one study used a survey [24] to collect data. The sample sizes of these studies ranged from 59 [16] to 52,689 [23]. Eight studies were on liver transplant, three on kidney transplant, and two on cardiac transplantation. Three studies primarily focused on cannabis use [13,23], one on injection drug use (IDU) [16], three studies did not present data on individual substances [18,24-25], and the others presented data on multiple substances.

**Table 1:** Studies reporting on the illicit drug use among solid-organ transplant candidates and those who received transplants ALD: alcoholic liver disease; HCV: hepatitis C virus, IDU: injecting drug use

Study/ year	Type of study	Sample size	Type of transplant	Results
Stark, (2019)	Retrospective cohort study	2067	Kidney	3% met criteria for cannabis abuse or dependence, 58% of them were daily users, 31% had other illicit drug dependencies
Schult, (2019)	Cross-sectional study, consecutive sample hospitalized 3 months after transplant	190	Liver	In the overall cohort, any illicit drug was used by 35% at some point in time, most frequently cannabis (31%), amphetamine (16%), heroin (5%), cocaine, inhalants, and hallucinogens (8% each)
Alhamed, (2019)	Database linking national kidney transplant records with Medicare claims	52,689	Kidney	Prevalence of cannabis abuse or dependence was diagnosed in 0.5% in the years before and (0.3%) after transplant
Kotwani, (2018)	Retrospective cohort of candidates listed for a liver transplant	884	Liver	Prevalence of cannabis use- 48%, recent use at the time of transplant evaluation- 7%, among marijuana users 13% had weekly use and 16% daily use 46% had a history of illicit drug use with 14% being recent users
Sacco, (2018)	Survey among liver transplant candidates with alcoholic liver disease	67	Liver	(47.8%) used illicit substances prior to transplant and 16.4% reported illicit drug use post-transplant
Tang, (2018)	Single‐center, retrospective, cohort study	2280	Kidney	Prevalence of current substance use- 17%, current tobacco use- 14%, current drug use- 5%; historical drug use 15%, majority of current (67%) or historical (54%) used cannabis
Lamba, (2012)	Cross-sectional descriptive using a mailed questionnaire	281	Liver	29% had a history of substance or alcohol use
Webzell, (2011)	Cross-sectional assessment of recent substance use among liver transplant candidates	109	Liver	Screen positivity was more common among patients with HCV (40%) and ALD (38%) versus patients with other liver diseases (18%). ALD patients most frequently had positive results for opiates (20%), benzodiazepines (15%). 20% of HCV patients had a positive screen for cannabis. Overall, ALD and HCV patients were more likely to have positive urine toxicology results (38%, 40%, and 18% positive, respectively), 4% of patients with ALD or HCV self-reported current alcohol or illicit drug use Positive urine toxicology screen in 30% of the participants. 29% of males and 33% of females had positive toxicological results 2% of patients had 3 substance use classes and 8% had 2 substance use classes
Gottardi, (2010)	Past injection drug users using data source of transplant registries of the University Hospitals	59	Liver	4.6% of transplanted patients had a history of IDU Everyone used heroin 14% also used cocaine and 13.6% also consumed other illicit drugs such as marijuana, LSD, 16.9% of the patients had a substitution therapy before and 6.8% continued substitution therapy after liver transplant
Ranney, (2009)	Retrospective cohort study	1489	Liver	10.4% (155 patients) were cannabis users during liver transplant evaluation period Marijuana users were mostly males and younger 31% of marijuana users also had a positive urine screen for narcotics, 21.9% had benzodiazepines, and 7.7% had other substances
Gedaly, (2008)	Retrospective chart review of alcoholic liver disease patients requiring a transplant	147	Liver	47.4% had previous or current use of one or more substances 17.2% continued to have objective evidence of illicit drug use post-transplant marijuana use amongst 28.5%, cocaine among 16.1%, IV drug use among 15.3%, and narcotic use among 17.5% of participants. Substance use after transplantation was marijuana 13.9%, cocaine 3.5%, and amphetamines 0.7% of the population
Hanrahan, (2001)	Retrospective data analysis	189	Heart	24.86% had a history of substance use pre-transplant
Shapiro, 1995	Prospective study	125 consecutive sample	Heart	20.3% with a past substance use history and 5.4% had a recent substance use history at the time of evaluation

Liver Transplant

The majority of the studies included in this review indicate that the most prevalent substance use among patients undergoing liver transplant is cannabis [15,22,24,26]. For example, Schult (2019) included liver transplant recipients with alcoholic liver cirrhosis (ALC), HCV, or Hepatocellular carcinoma (HCC) but without ALC and patients with other liver diseases [26]. The most frequently used illicit substance was cannabis (31%), followed by amphetamine (16%), heroin (5%), cocaine, inhalants, and hallucinogens (8% each) [26]. Almost 50% of the overall cohort used prescription narcotic drugs, and 35% used at least one illicit drug at some point in time in their life [26]. Similarly, in another study, the prevalence of marijuana use was 48%, with 7% recent user (defiant as self-reported ongoing marijuana use at the time of first liver transplant evaluation and/or had positive drug toxicology on screening) among patients listed for liver transplantation [15]. Sacco et al. (2018), in a survey among liver transplant candidates with alcoholic liver disease, found that nearly half of participants (47.8%) used illicit substances before transplant, and 16.4% reported illicit drug use post-transplant [24]. Fifty-nine percent of those who reported substance use in the pre-transplant period reported polysubstance use, with cannabis being the most common (48%) form of illicit substance use, closely followed by opioid use (46%). The other substance uses reported in the pre-transplant period were cocaine and sedatives (34% and 22% of those who were using substances, respectively. Substance use lowered significantly in the post-transplant period, especially the non-cannabis substances. Similarly, in another study by Gedaly et al. (2008), among liver transplant recipients for alcoholic liver disease, 47.4% of the participants had previous or current use of one or more substances and 17.2% continued to have objective evidence of illicit drug use after transplant [22]. Cannabis use was found amongst 28.5%, cocaine among 16.1%, injectable drug use among 15.3%, and narcotic use among 17.5% of participants. Substance use after transplantation included marijuana in 13.9%, cocaine in 3.5%, and amphetamines in 0.7% of the population [22].

Other studies concluded lower rates of substance use among transplant recipients [10,25]. Lamba et al. (2012) reported alcohol and substance use history among one-third of liver transplant recipients [25]. The authors did not provide any details on the types of substances or the rates in each category. A study conducted by Webzell et al. (2011) also found a similar (30%) rate of substance use among adults admitted for liver transplant assessment [10]. Two percent of the patients included in Webzell’s study (2011) had three substance use classes, and 8% had two substance use classes. Screen positivity was more common among patients with HCV (40%) and those with alcoholic liver disease (ALD) (38%) as compared to patients with other liver diseases (18%). ALD patients most frequently had positive results for opiates (20%) and benzodiazepines (15%). About 20% of HCV patients had a positive screen for cannabis. Overall, ALD and HCV patients were more likely to have positive urine toxicology results (38%, 40%, and 18% positive, respectively), and 4% of patients with ALD or HCV self-reported current alcohol or illicit drug use [10]. Ranney (2009) investigated marijuana use among individuals evaluated for liver-transplantation patients [17]. Among 1489 patients included in their study, they found out marijuana use in only 155 individuals (10%). In this retrospective chart review, authors found out that 43 (27%) of marijuana users were listed whereas, 593 (44%) of non-users were listed for transplantation. The authors excluded those who self-reported marijuana use but had a negative urine toxicology screen. While the authors attempted to be more objective in identifying cannabis use but potentially excluded those with relatively lower severity of use, leading to a lower rate of marijuana use among liver-transplant candidates. Twenty-one point zero five percent (21.05%) had a positive narcotic screen, 11.23% had a positive benzodiazepine screen, and 3.12% had positive other substance screens [17].

Kanchana (2002) reviewed the chart of orthotopic liver transplant recipients of their center and found 2.7% of transplant recipients had a history of heroin use and had undergone treatment for alcohol and substance use but could not be weaned off from methadone [21].

We found only one study that exclusively investigated injecting drug users among liver-transplant recipients [16]. De Gottardi et al. (2010) identified past injection drug users amongst liver-transplant recipients from the transplant registries of the University Hospitals of Geneva and Lausanne (Switzerland) [16]. The reported prevalence of injecting drug users (IDU) was 4.6% among transplanted patients. Such a low rate could be related to the selection process of transplant candidates that often excludes people who inject drugs from the transplant list [16]. All of the injecting drug users used heroin, 14% also consumed cocaine, and 13.6% consumed other illicit substances such as marijuana, LSD, or opium. Amongst the injecting drug users, 16.9% had a history of substitution therapy and 6.8% continued substitution therapy after liver transplant. About 3.4% of the 59 patients with a history of IDU relapsed using injection drugs over a 51-month mean follow-up period. The authors concluded that documented IDU was rare in liver transplant patients.

Kidney Transplant

We found three studies that investigated the prevalence of substance use among kidney transplant recipients. The reported prevalence appeared to be relatively less as compared to liver transplant candidates. This difference could be due to the difference in data collection method, the difference in candidate selection, and/or an actual lower rate of substance use among kidney transplant candidates as compared to liver transplant candidates. However, it should be kept in mind that we included only three studies in this systematic research, although each had a decent sample size.

In a study by Stark et al. (2019), 3% of those referred by social workers to the addiction psychiatrist met the criteria for cannabis abuse or dependence (CAD) [13]. Amongst the cannabis users, 58% consumed cannabis daily, 47% had a history of alcohol dependence, and 31% also had other substance use history [13]. The low rate of marijuana and other substance use could be due to the fact that abstinence was actively encouraged for listing. Similarly, in another study, the prevalence of cannabis abuse or dependence was diagnosed among 0.5% of the study population in the years before and 0.3% after transplant [23]. The low rate reported in Alhamad’s (2019) study could be because the study population was the Medicare population who were already selected to undergo transplantation [23]. The authors (Alhamed) suspected that those with substance abuse behaviors (that would preclude transplant) were not included in the study, and the diverse coding practice among various treatment providers did not follow the usual rigor that is typically conducted in clinical trials and researches. Similarly, Tang et al. (2019) found out that the prevalence of any substance use was 17%, current drug use was 5%, and 67% of the current drug users were cannabis users [14]. About 15% had historical drug use, with 54% had cannabis use among historic drug users [14].

Heart Transplant

We found only two studies carried out on cardiac transplant patients. In a study by Hanrahan et al. (2001), in a pool of 189 heart transplant recipients, 47 (24.86%) had a history of alcohol and substance use [18]. The authors did not separate alcohol from other forms of substance use and clustered everyone in one category. Authors did not indicate if everyone were current use or if this was lifetime use history and if laboratory measures were also part of the assessment of substance use history. The results from the other study on cardiac transplant patients [19], involving 125 consecutive adults receiving cardiac transplantation, found 20.3% with a past history of substance abuse, and 5.4% had a recent substance use history at the time of evaluation for cardiac transplantation. It is unclear if the authors included both alcohol use and other illicit substance use under the same category of substance use.

Lung Transplant

We could not find any study that is directly addressing the research question on substance use and lung transplantation.

Our research indicates that substance use in some form is present in almost half the solid-organ transplantation candidates, especially when assessed at the time of ongoing pre-transplant evaluation. Cannabis appears to be the most common illicit substance use among solid-organ transplantation recipients. The other substances used by solid-organ transplant recipients are opioids, cocaine, and benzodiazepines, but the data are sparse.

The implications of illicit drug use on the outcome of solid-organ transplant

Eleven studies reported the outcomes of transplant recipients (Table 2). Eight of them analyzed retrospective data [8,15-18,20-21,27], one study used national transplant data [23], one study used a mailed questionnaire [25], and one used a prospective design [19]. The sample sizes of these studies ranged from 27 [8] to 52,689 [23]. Seven studies were conducted among liver transplant candidates [8,15-17,20-21,25], two studies were done in kidney recipients [23,27], and two on heart recipients [18-19]. Three studies focused primarily on cannabis [17,23,27], two focused on IDU [16,20], and one study was exclusively for those who are on methadone maintenance [21].

**Table 2:** Relationship of illicit drug use and outcome among transplant recipients CI: confidence intervals; HR: hazards ratio; IDU: injecting drug use

Author/ Year	Type of study	Sample size	Type of transplant	Outcome
Alhamed, (2019)	Analyzing database linking national kidney transplant records with Medicare claims	52,689	Kidney	Cannabis abuse or dependence in the year before the transplant was not associated with death or graft failure in the year after transplant Cannabis abuse or dependent in the pre-transplant period was associated with post-transplant alcohol and other drug use, non- compliance, schizophrenia and depression, mental status changes, delusions. Post-transplant cannabis use was also associated with graft loss
Kotwani, (2018)	Retrospective cohort of candidates listed for Liver transplant	884	Liver	Recent illicit drug use was associated with a higher risk of death or delisting Unlike illicit drug use, marijuana use was not associated with worse outcome among liver transplant waitlist candidates
Greenan, (2016)	Retrospective chart review	1225	Kidney	Isolated recreational marijuana use is not associated with poorer patient or kidney allograft outcomes at 1 year
Lamba, (2012)	Cross-sectional descriptive using a mailed questionnaire	281	Liver	Treatment non-adherence rate was higher than those with no history (61% vs 46%) of alcohol or drug use
Gottardi, (2010)	Analysis of data of individuals with a past history of IDU from transplantation registries	59	Liver	Past IDU not associated with poorer survival after liver transplant Relapse rate after liver transplant occurred in 3.4%
Ranney, (2009)	Retrospective cohort study	2007	Liver	Hepatitis C and transplantation were associated with survival, but not marijuana use was not (HR 1.09, 95% CI 0.78-1.54). A similar survival rate irrespective of marijuana use did not pose any additional risk of mortality among liver transplant patients
Robaeys, (2009)	Retrospective chart review of Chronic Hepatitis C patients	67	Liver	Same compliance and patient and graft survival among patients in the non-IDU group, and patients with chronic hepatitis C infected after IDU
Nickels, (2007)	Retrospective chart review of post-transplant recipients with pre-transplant polysubstance use	27	Liver	Rate of recidivism was 26.9% No effect on survival because of relapse to substance use
Kanchana, (2002)	Review of liver transplant recipient data	185	Liver	5 transplant recipients (2.7%) had a history of heroin abuse and could not be weaned off from methadone maintenance treatment and had no negative effect on transplant outcome
Hanrahan, (2001)	Retrospective data analysis	189	Heart	The elevated rate of heart-related causes of death, noncompliance, and death related to noncompliance but no significant difference in the overall survival rate between substance users vs. non-substance user group
Shapiro, (1995)	Prospective study	125 consecutive sample	Heart	Treatment non-adherence was associated with substance use

The outcome research of solid-organ transplant and illicit drug use has investigated multiple domains, making it nearly impossible to consolidate in a few broad areas. The included studies showed that substance use is associated with a higher risk of delisting [15], alcohol and other drug use [23], schizophrenia [23], depression [23], idiopathic cardiomyopathy and heart-related causes of death [18] in context to various solid-organ transplants. However, there are two important areas that most of the included studies have commented upon is worth mentioning. One is the association of illicit drug use and treatment compliance and survival after transplant.

The review indicates that the recipient's substance use adversely affects the medication non-adherence. A better outcome of solid-organ transplant requires strict adherence to the treatment regimen, keeping follow-up appointments with healthcare providers, and bringing a significant change of lifestyle and behavioral modification [28]. Non-compliance, in turn, is often associated with late acute rejection, graft loss, and death [29]. In a study by Lamba et al. (2012), 29% of their sample size had a history of substance or alcohol use, and the treatment non-adherence rate was higher than those with no such history (61% vs. 46%) [25]. However, the study population was from an urban public hospital, and the survey instrument was not the “gold standard.” In a retrospective chart review in patients with chronic hepatitis C who underwent a liver transplant, seven (10%) patients with and 60 patients without a history of IVDU were compared, and the authors did not find any statistically significant difference in between the groups in terms of compliance and between patient and graft survival after liver transplantation [20]. The survival rates at the end of the first, third, and fifth years were 86%, 84%, and 81%, respectively, in the non-IVDU group, which is similar to 84% at each of the time periods in the IVDU group. A similar outcome was also found in another study conducted using data of liver transplant candidates from Switzerland and France between 1988 and 2006 among 59 patients with a history of IDU [16]. In this study, 16.9% of patients had substitution therapy before and 6.8% continued opioid substitution therapy following a liver transplant [16]. This study found that IDU was rare in liver transplant patients, and past IDU was not associated with poor survival after liver transplant. A study conducted by Kotwani et al. (2018), among candidates listed for liver transplantation, showed that recent illicit drug except marijuana use was associated with a higher risk of death or delisting among liver transplant waitlist candidates over a period of two years [15].

Similarly, in a retrospective cohort study, Ranney et al. (2009) found out that hepatitis C was poorly associated with survival after liver transplantation while marijuana use was not (HR 1.09, 95% CI 0.78-1.54) [17]. But, at the same time, a significantly higher proportion of marijuana users were associated with hepatitis C infection than non- users. The authors concluded that patients with marijuana use did not have any higher hazard of mortality among liver transplant patients. Alhamad’s research (2019) also indicates that cannabis abuse or dependence in the year before the transplant was not associated with death or graft failure in the year after transplant, but it was associated with post-transplant issues such as alcohol and other drug use, non-compliance, schizophrenia and depression, mental status changes, and delusions [23]. The post-transplant cannabis use was also associated with graft loss even after adjusting the alcohol use, other substance use, and noncompliance.

De Gottardi et al. (2010) did not find any association of past injection drug use with poorer survival after liver transplant over an average follow-up of 51 months [16]. The rate of relapse into IDU after transplant was also rare. The post-transplant survival also did not depend upon whether or not the patient was on methadone substitution therapy. A similar result was found in a study by Kanchana et al. (2002) on the outcome of orthotopic liver transplantation in patients with end-stage liver disease who were on methadone maintenance therapy [21]. This study is limited by the sample size that precludes the generalization of the finding. The researchers presented cases of five patients with end-stage liver disease who underwent a liver transplant and had a history of heroin use and could not be weaned off methadone [21]. The researchers did not find any adverse effect on the outcome of liver transplant recipients on methadone maintenance therapy, and the authors suggested that cirrhotic patients on methadone maintenance therapy should be considered for a liver transplant if they met the same psychosocial requirements as patients with alcohol abuse, and they do not need to be weaned off methadone before liver transplant.

Nickels et al. (2007) retrospectively reviewed the record of post-transplant patients referred to transplant psychiatrists. The psychiatrist identified post-transplant patients with more than one substance abuse pre-transplant but not exclusively alcohol abuse or dependence [8]. Over a span of five years, their center catered to 27 patients with more than one substance use, who received a liver transplant for end-stage liver disease. The researchers assessed patient survival by means of an existing survival database. The authors did not find any difference in one-year survival between relapsed and non-relapsed groups. The rate of recidivism was 26.9% [8]. The study was limited by low sample size. There was also a selection bias, as the study included only those who were referred to the transplant psychiatrist.

Only two studies examined the effect of substance use on cardiac transplants. Both studies support the association of substance use history with non-adherence to post-transplant treatment. In a 10-year prospective study, Hanrahan (2001) found a higher rate of idiopathic cardiomyopathy, heart-related cause of death, noncompliance, and death-related noncompliance among those with a substance use history as compared to those without a substance use history and receiving cardiac transplantation [18]. However, the researchers did not find any significant difference in the overall survival rate between the substance users vs. non-substance user groups. The other study about cardiac transplant patients by Shapiro et al. (1995) involved 125 consecutive adults who received cardiac transplantation over a 13.8 ±9.9-month period showed that the treatment non-adherence was associated with a substance abuse history, personality disorder, living arrangements, and global psychosocial risk [19].

Greenan et al. (2016) retrospectively reviewed 1225 kidney recipients from 2008 to 2013 [27]. Marijuana use was defined as a positive urine screen and or self-reported recent use. The authors compared outcomes between marijuana users and non-users and did not find out any worse primary outcome (death at one year or graft failure, defined as glomerular filtration rate <20 mL/min/1.73 m^2^) between users vs. non-users using logistic regression analysis. The authors concluded that isolated recreational marijuana use was not associated with the poorer patient or kidney allograft outcomes at one year [27].

Discussion

Overall, it seems like pre-transplant illicit drug use affects various aspects of the outcome of solid-organ transplants. However, a general conclusion is challenging, as there is no uniformity in the outcome measures used by the included studies and a general statement on the risk of substance use on the outcome of a solid-organ transplant cannot be made. It appears that the bulk of the literature indicates that substance use is associated with poor treatment adherence, which is associated with various other adversities, but it probably does not affect the overall survival rate, particularly in the case of cannabis use. The majority of the studies addressing survival did not find any significant adverse effects of pre-transplant marijuana use on survival [15,17,23,27] among liver or kidney transplants. Studies showing no significant effect of IDU on survival [16,20] are limited by low sample sizes, a finding that warrants replication studies involving larger sample sizes. However, substance use is associated with poor treatment adherence [18-19,25] in the post-transplant period, which is associated with late acute rejection, graft loss, and death [29]. Limited data support that polysubstance use in liver transplant did not affect survival but has a significant rate of recidivism [8]. Only one study with a limited sample size indicates that those who are on methadone maintenance therapy may not need to be weaned off before transplant and can safely continue maintenance therapy. Clearly, no generalization can be made based on such a small study, but if the results can be confirmed by replication studies involving larger sample sizes, it can potentially enhance access to the transplantation service of those who are on methadone maintenance treatment. This is especially true given that only about half of transplant programs accept patients who were on methadone maintenance [30]. There is a paucity of outcome data on the heart, pancreas, lung, and other solid-organ transplants, and a generalization cannot be made at this time.

This review highlights several aspects of illicit drug use, which are pertinent to solid-organ transplants. First, given the high prevalence of substance use among individuals needing a solid-organ transplant, excluding patients based on a history of substance use means depriving a substantial portion of individuals who will otherwise be benefited by a solid-organ transplant. Second, despite some challenges, such as treatment non-adherence and the risk of recidivism, there is possibly a similar survival rate among individuals with illicit drug use and those without a history of illicit drug use. Third, there is a need for further exploration of the topic of the safety of the continuation of methadone maintenance therapy in pre and post-solid-organ transplant candidates.

The interpretation of our review should be used with caution. Inclusion of the journal articles that are indexed in PubMed may not have captured some other available studies on this topic. The studies included in this review are significantly dissimilar, with some looking at individual substances like marijuana and cocaine, while others looking at a combination of multiple substances. With these limited data available, involving such a heterogeneous study population and study design, it is very challenging to draw a sweeping conclusion compiling these studies. All the studies included in this review focused on a single type of solid-organ transplant, and the majority of the data was from liver transplants followed by kidney transplants. The difference in the sampling method and the lack of universally accepted inclusion criteria for transplant recipients could account for the variation in the prevalence of substance use among transplant candidates. This shortcoming precluded us from generating more quantitative data on the prevalence of illicit substance use pre and post-transplant candidates.

Despite the limitations, the findings of the study have possible implications for clinicians in the context of solid-organ transplantation. Since patients with illicit substance use may have significant non-adherence to medications and worse outcomes, patients with a history of drug use should be carefully assessed and followed up, and treatment should be provided. One needs to consider that the patients may not volunteer a history of psychoactive substance use, especially when it is likely to be appraised negatively by the treating clinicians. In such circumstances, toxicological screening with non-invasive methods might be helpful for better patient selection.

## Conclusions

To our knowledge, this is the first review solely dedicated to illicit substance use in the context of solid organ transplantation. We aimed at identifying data on the prevalence of illicit substance use in the pre and post-transplant period and the effect of the history of illicit substance use on the outcome of solid organ transplantation. Cannabis appears to be the most common form of substance use in the context of solid organ transplant and its reported prevalence is up to 50% among studies. A more accurate measurement was not possible due to the heterogeneity of the included studies. The other conclusion is that substance use is associated with worse treatment adherence but may not affect survival following a solid organ transplant, especially in the context of cannabis use. However, in the future, studies can look at whether potential recipients cease substance-taking behavior; and whether and for how long are recipients able to prevent a lapse or relapse to illicit substance use. Further studies can look into illicit substance use among cardiac, pancreas, and lung transplantation, for which data is extremely limited. Studies can also look at whether illicit substance use interacts with behavioral markers like adherence to predict the quality of life, survival, and graft rejection. In addition, data about the concurrence of self-report and biochemical testing can help in identifying illicit substance use among potential recipients, and transplant cases can help clinicians make informed decisions.

## Figures and Tables

**Figure 1:**
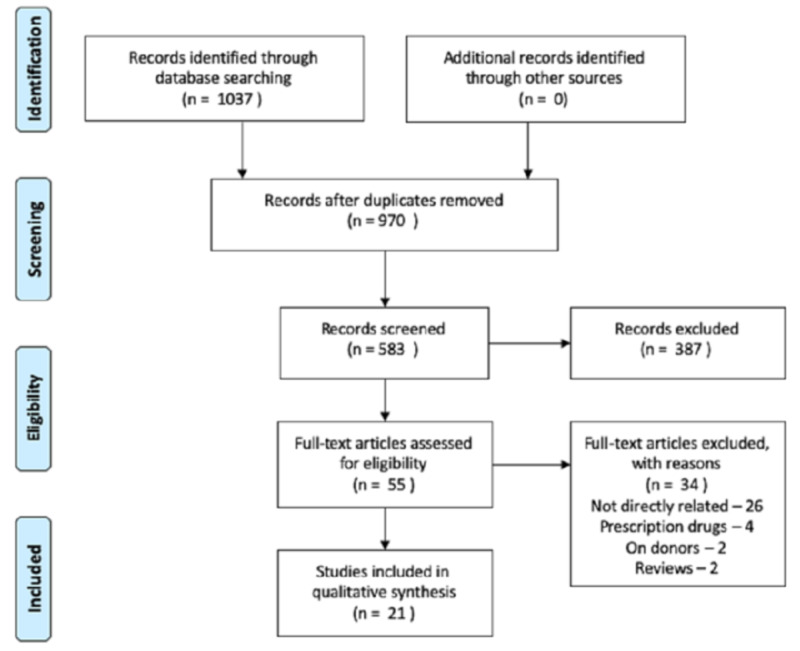
PRISMA flowchart PRISMA: Preferred Reporting Items for Systematic Reviews and Meta-Analyses Source: Moher D, Liberati A, Tetzlaff J, Altman DG [31] For more information, visit www.prisma-statement.org
